# Probabalistic reinforcement learning impairments predict negative symptom severity and risk for conversion in youth at clinical high-risk for psychosis

**DOI:** 10.1017/S0033291724003416

**Published:** 2025-02-06

**Authors:** Lauren Luther, Ian M. Raugh, Gregory P. Strauss

**Affiliations:** 1 Department of Psychology, University of Alabama at Birmingham, Birmingham, AL, USA; 2 Department of Psychology, University of Georgia, Athens, GA, USA; 3 Department of Psychiatry, Douglas Mental Health Institute, McGill University, Montréal, QC, Canada

**Keywords:** anhedonia, asociality, attenuated psychosis syndrome, avolition, reward learning, ultra-high-risk

## Abstract

**Background:**

Elucidation of transphasic mechanisms (i.e., mechanisms that occur across illness phases) underlying negative symptoms could inform early intervention and prevention efforts and additionally identify treatment targets that could be effective regardless of illness stage. This study examined whether a key reinforcement learning behavioral pattern characterized by reduced difficulty learning from rewards that have been found to underlie negative symptoms in those with a schizophrenia diagnosis also contributes to negative symptoms in those at clinical high-risk (CHR) for psychosis.

**Methods:**

CHR youth (*n* = 46) and 51 healthy controls (CN) completed an explicit reinforcement learning task with two phases. During the acquisition phase, participants learned to select between pairs of stimuli probabilistically reinforced with feedback indicating receipt of monetary gains or avoidance of losses. Following training, the transfer phase required participants to select between pairs of previously presented stimuli during the acquisition phase and novel stimuli without receiving feedback. These test phase pairings allowed for inferences about the contributions of prediction error and value representation mechanisms to reinforcement learning deficits.

**Results:**

In acquisition, CHR participants displayed impaired learning from gains specifically that were associated with greater negative symptom severity. Transfer performance indicated these acquisition deficits were largely driven by value representation deficits. In addition to negative symptoms, this profile of deficits was associated with a greater risk of conversion to psychosis and lower functioning.

**Conclusions:**

Impairments in positive reinforcement learning, specifically effectively representing reward value, may be an important transphasic mechanism of negative symptoms and a marker of psychosis liability.

## Introduction

Psychotic disorders (PD) are severe and debilitating mental illnesses that incur suffering, lead to high rates of functional disability, and present challenges for the healthcare system (Harvey & Strassing, 2012; Kadakia et al., 2022). Given that few individuals achieve recovery after the onset of PDs, there has been increased interest in the early identification and prevention of psychosis. PDs are often preceded by a prodromal (i.e., pre-illness) period characterized by functional decline and attenuated hallucinations and delusions that progressively worsen over time (Addington & Heinssen, 2012). This period not only reflects a potential point of early intervention but also a critical window for investigating processes related to illness onset. The field’s current approach to identifying processes giving rise to psychosis has focused on tracking pluripotent (i.e., general psychopathology) risk factors, stable traits of PDs (e.g., cognitive deficits), and stable vulnerability markers that also occur in unaffected relatives (Addington, Farris, Devoe, & Metzak, 2020; Cannon, 2005; Cannon et al., 2000). Although this approach has led to important advances, it has inherently resulted in poor specificity, a high false-positive rate, and not identified core pathophysiological processes that could serve as treatment targets (Fusar-Poli et al., 2020; Gold et al., 2020). To make progress in preventing the onset of PDs and the functional disability that accompanies them, the field needs novel approaches to identifying candidate mechanisms.

We propose that a novel approach to addressing this need involves investigating mechanisms underlying negative symptoms. There are several reasons why this alternative approach may be beneficial. First, among those at clinical high-risk (CHR) for psychosis, negative symptoms are one of the strongest predictors of functional outcome (Devoe, Braun, Seredynski, & Addington, 2020; Minichino et al., 2017; Schlosser et al., 2015), suggesting that a better understanding of their mechanisms has the potential to lead to new treatments. Second, negative symptoms are highly prevalent in the prodromal phase (Piskulic et al., 2012) and one of the earliest indicators of risk that emerge years before attenuated positive symptoms (Addington & Heinssen, 2012). Third, negative symptoms are often the reason why CHR youth and their families make initial contact with the treatment system (Yung & McGorry, 1996) and are one of the strongest predictors of conversion (Alderman et al., 2015; Demjaha et al., 2012; Healey et al., 2018). These findings suggest that identifying mechanistic processes underlying negative symptoms has the potential to shift the identification of psychosis to earlier stages than has been possible, which is important because the majority of functional decline has already occurred by the time attenuated positive symptoms are present (Fusar-Poli et al., 2013). In particular, the volitional dimension of negative symptoms, as opposed to the expressive dimension, is highly predictive of poor functional outcomes in CHR (Pelletier-Baldelli, Strauss, Visser, & Mittal, 2017; Strauss et al., 2023). Although mechanisms underlying the volitional dimension have been identified in adults with PDs (Marder & Umbricht, 2023; Sarkar, 2015), little research has examined whether these same mechanisms also predict motivational symptoms in CHR.

Influential conceptual models developed for adults with PDs propose that volitional negative symptoms result from dysfunctional cortico-striatal circuitry and abnormalities in reward processing that impact the decision-making processes needed to initiate goal-directed activities (Kring & Barch, 2014; Strauss, Waltz, & Gold, 2014). One key reward processing area identified as being impaired in PDs and linked to negative symptoms is reinforcement learning (RL) (Waltz, Frank, Wiecki, & Gold, 2011; Waltz & Gold, 2007), which involves learning which stimuli and behaviors are linked to reward outcomes. Two interactive and complementary neural systems support RL. The first is mediated by the prefrontal cortex and involves rapid learning processes, such as the ability to update value representations on a trial-by-trial basis using reward feedback (Frank & Claus, 2006; Gonzalez et al., 2013). This system is critical for facilitating goal-directed action because it allows individuals to respond to changes in reinforcement contingencies within the environment. The second system, which is mediated by the basal ganglia, governs gradual reinforcement learning that occurs over a number of trials (Frank, Loughry, & O’Reilly, 2001; Hélie, Ell, & Ashby, 2015; Packard & Knowlton, 2002). Critically, both the fast and slow RL systems make use of prediction errors (PEs), which occur when there are mismatches between obtained and expected reward outcomes. PEs are divided into those that are positive (i.e., transient bursts of dopamine cell activity that occur for better than expected outcomes) and negative (i.e., transient cessations in dopamine cell activity that occur when outcomes are worse than expected) (Cohen & Frank, 2009; Schultz, 1998).

Several studies have investigated the integrity of the rapid and gradual RL systems in PDs and their associations with negative symptoms. Individuals with PDs have consistently demonstrated impairments in rapid learning on explicit RL tasks, consistent with deficits in making adjustments in decision-making based on trial-by-trial feedback (Barch et al., 2017; Cheng et al., 2012; Cicero, Martin, Becker, & Kerns, 2014; Gold et al., 2012; Koch et al., 2010; Reinen et al., 2016; Strauss et al., 2014; Waltz, Frank, Robinson, & Gold, 2007; Yilmaz, Şimşek, & Gonul, 2012). These rapid learning deficits are associated with reduced activation of the orbitofrontal cortex and greater negative symptom severity (Strauss et al., 2011; Waltz et al., 2007, 2011). In contrast, more gradual implicit RL governed by the basal ganglia appears intact in PDs (Collins et al., 2014, 2017); however, recruitment of regions other than the basal ganglia has been demonstrated, indicating that normal implicit RL may be accompanied by aberrant patterns of compensation (AhnAllen et al., 2012; Barch et al., 2017; Heerey, Bell-Warren, & Gold, 2008; Pratt et al., 2021; Reiss et al., 2006; Weickert et al., 2009). Furthermore, RL paradigms that manipulate positive and negative feedback have shown that individuals with PDs typically have intact learning from negative feedback but impaired learning from positive feedback (Cheng et al., 2012; Gold et al., 2012; Reinen et al., 2014; Strauss et al., 2011; Waltz et al., 2007). This pattern of performance has been specifically associated with greater volitional negative symptom severity and can be considered a neurobehavioral recipe for reduced goal-directed activity (i.e., individuals are capable of learning what not to do to avoid negative outcomes but not what to do to obtain rewards) (Gold et al., 2012; Reinen et al., 2014; Strauss et al., 2011, 2014; Waltz et al., 2007, 2011). Multiple processes may contribute to impaired learning from positive feedback in PDs. For example, consistent with aberrant positive prediction error signaling, neuroimaging studies have shown that reduced activation of the ventral striatum occurs in response to reward outcomes and that these abnormalities are associated with greater negative symptoms (Culbreth et al., 2020; Prettyman et al., 2021; Wolf et al., 2014). Alternatively, computational modeling studies that are capable of simulating the effects of the basal ganglia and orbitofrontal cortex suggest that prediction error signaling may be intact and that poor learning from positive feedback results from orbitofrontal cortex-driven deficits in value representation (Strauss et al., 2011; Waltz & Gold, 2015).

Despite their importance, few studies have examined RL mechanisms underlying negative symptoms in CHR. Like PDs, there is evidence for intact implicit RL in CHR on tasks relying on gradual learning and basal ganglia-driven mechanisms (Spilka et al., 2023); however, unlike PDs, individual differences in performance in gradual reinforcement learning are associated with negative symptom severity in CHR (Spilka et al., 2023). Studies examining rapid RL and learning from positive versus negative feedback on explicit RL tasks have produced more mixed results. For example, there is some evidence for deficits in rapid RL and impaired learning from gains that are associated with greater negative symptoms and blunted prediction error signals in the ventral striatum, dorsal anterior cingulate cortex, and ventromedial prefrontal cortex (Millman et al., 2020; Schmidt et al., 2017; Waltz et al., 2015). However, other studies have reported intact explicit RL (Strauss et al., 2021). Furthermore, aberrant prediction error signaling in the dorsal anterior cingulate cortex and explicit RL deficits have also been associated with greater severity of depression (Millman et al., 2020), calling into question whether associations between RL and negative symptoms reflect secondary processes due to mood diagnoses that are highly comorbid in CHR (Fusar-Poli et al., 2014). Thus, it is currently unclear whether RL deficits play the same role in CHR in the development and maintenance of negative symptoms as they do in PDs.

To evaluate the nature of RL and its association with negative symptoms in CHR, the current study administered an explicit probabilistic RL task (PRLT) previously used in PDs to identify explicit RL components linked to negative symptoms (Gold et al., 2012). The paradigm consists of two task phases. The first phase is termed acquisition and involves simultaneously learning 4 stimulus pairs over a number of trials. Two of the stimulus pairs assess gain learning, where the correct choice leads to a probabilistically reinforced monetary reward on 90% or 80% of trials, and incorrect choices lead to no reward. The other 2 pairs assess loss avoidance, where the correct choice allows for the avoidance of a monetary loss on 90% or 80% of trials, and an incorrect response results in a monetary loss. Importantly, both the gain and loss avoidance pairs generally result in a positive prediction error when participants select the optimal response (i.e., gain reward or avoid losing reward, respectively). This manipulation is critical for the second portion of the task, called the transfer phase, where participants select between two stimuli in the absence of feedback. Stimuli presented in the acquisition phase are presented along with novel stimuli, allowing for critical pairings such as the frequent winner versus frequent loss avoider (i.e., both stimuli are associated with positive prediction errors but only the frequent winner requires representation of positive value). Additional pairings allow for an enhanced understanding of the role of prediction error and value representation processes contributing to RL impairments: frequent winners versus infrequent winners, frequent winners versus frequent losers, and frequent loss avoiders versus infrequent winners. Past PD studies using this task have found evidence for impaired rapid learning and a selective deficit in learning from gains but intact loss avoidance learning during the acquisition phase (Gold, 2012; Reinen et al., 2014; Waltz et al., 2007, 2011). In the transfer phase, individuals with a PD displayed a failure to select the frequent winner over the frequent loss avoider, consistent with a deficit in representing reward value rather than prediction error signaling (Gold, 2012). Furthermore, greater negative symptoms were associated with reduced learning rate, impaired learning from gains, better loss avoidance learning, and impaired selection of the frequent winner over the frequent loss avoider in the transfer phase (Gold et al., 2012; Reinen et al., 2014; Waltz et al., 2007, 2011). Based on these past studies, it was hypothesized that relative to healthy controls (CN), CHR participants would display: (1) reduced learning in the acquisition phase, consistent with impaired rapid learning; (2) impaired learning from gains, but intact loss avoidance learning; (3) deficits in value representation, as reflected in transfer phase behavior and the failure to select frequent winners over frequent loss avoiders. Additionally, the patterns in hypotheses 1–3 were expected to significantly correlate with motivational negative symptoms.

## Methods

### Participants

Participants included 46 CHR participants and 51 CN. CHR participants were recruited from the Georgia Psychiatric Risk Evaluation Program (G-PREP) and the New York Psychosis Risk Evaluation Program (NY-PREP). These programs perform diagnostic evaluations for youth displaying prodromal syndromes. Participants were also recruited through online and printed advertisements and through presentations or meetings with members of community mental health centers and local school systems. CHR participants met the criteria for a prodromal syndrome on the Structured Interview for Prodromal Syndromes (SIPS) (Miller et al., 2003) (see Supplementary Material for details).

CN were recruited through online and printed advertisements. CN were eligible if they had no current major psychiatric disorder diagnosis or schizophrenia-spectrum personality disorder as established on the SCID-5 (First, Williams, Karg, et al., 2015) and SCID-5-PD (First, Williams, Benjamin, & Spitzer, 2015). CN also had no family history of psychosis and were not taking psychotropic medications. Groups did not significantly differ in age, sex, parental education, or race (See Table 1). CHR had lower education than CN.

**Table 1.** Sample characteristics

Variable	CHR(*n* = 46)	CN(*n* = 51)	Test Statistic	*p*
Age; M (SD)	20.39 (2.44)	20.42 (2.02)	*t* = .06	.95
Male; *n* (%)	11 (24%)	13 (25%)	χ^2^ = .97	.62
Personal education	13.50 (1.64)	14.22 (1.61)	*t* = 2.17	.03
Parental education	15.16 (2.60)	15.30 (2.09)	*t* = .28	.78
Race	n (%)	n (%)	χ^2^ = 1.83	.77
African American	3 (6%)	2 (4%)		
Asian American	6 (13%)	8 (16%)		
Caucasian	32 (70%)	37 (74%)		
Hispanic/Latino	4 (9%)	3 (6%)		
Other	1 (2%)	0 (0%)		

*Note:* One healthy control was missing race/ethnicity. CHR, clinical high-risk group; CN, healthy control group; M, mean; SD, standard deviation.

### Procedures

After providing informed consent, participants completed clinical interviews. All participants were rated on the SCID-5. CN also completed the SCID-5-PD, and CHR was rated on the SIPS and the Brief Negative Symptom Scale (Kirkpatrick et al., 2011), Clinical High-Risk Adaptation Version (Strauss & Chapman, 2018). The positive symptoms SIPS subscale was used to measure positive symptoms, the SIPS dysphoric mood item was used as an index of depression, and the Global Assessment of Functioning (Hall, 1995) modified scale from the SIPS was the measure of functioning. After completion of the rating scales, all participants completed the PRLT. Participants were compensated for study procedures at a rate of $30 per hour and received a PRLT task bonus reflecting a proportion of their monetary task winnings of $5.00. Participants provided written informed consent for a protocol approved by the University of Georgia or Binghamton University Institutional Review Boards.

For CHR participants, Zhang et al. (2018)’s Shanghai at Risk for Psychosis (SHARP) cross-sectional conversion risk score was calculated. The SHARP risk calculator is a tool that gives a participant’s cross-sectional probability of converting to a formal psychotic disorder at any point in time. It is derived based on clinical variables from the SIPS that are most predictive of conversion to full psychosis, with an algorithm that weighs functional decline, positive symptoms, negative symptoms, and dysphoric mood. Higher scores on this risk calculator significantly predicted conversion probability in the original Shanghai development and validation samples (Zhang et al., 2018) as well as in independent replications (Osborne & Mittal, 2019).

### Measures

The Probabilistic Reinforcement Learning Task (PRLT) is based on Frank, Seeberger, & O’Reilly, 2004; Gold et al., 2012. In the acquisition phase, participants were asked to learn how four pairs of stimuli were associated with monetary gains or loss avoidance. Stimuli in this task were landscape images (see Supplementary Material), and each pair was presented separately. Two of the stimuli were associated with potential gains. Choosing the correct stimulus was probabilistically reinforced with the monetary outcome (winning money is indicated by a coin along with the word “win”). If the incorrect stimulus was chosen, participants received no reward, which was indicated by seeing the phrase “Not a winner, try again.” One of the stimulus pairs reinforced the correct response on 90% of trials, while the other pair reinforced the correct response on 80% of trials. The other two stimuli pairs required participants to learn to avoid losses. The correct response was probabilistically associated with not losing money (the feedback of “Keep your money” is shown), while the incorrect response was probabilistically associated with losing money (feedback of “Lose” was presented along with a coin with an X through it). The correct response was associated with avoiding losses on 90% of trials in one pair and 80% of trials in the other pair. After a practice session, participants completed 160 learning trials with the four stimulus pairs presented in a randomized order. Each pair was shown 40 times, and the 160 trials were divided into 4 blocks of 40 trials for analyses.

The transfer phase consisted of 64 trials where the original four stimulus pairs from the learning phase were shown 4 times. In addition, 24 novel pairings of the stimuli used in the learning phase were presented twice. Novel pairings consisted of a trained item from the acquisition phase presented with every other trained item (e.g., a stimulus from the 90% gain learning was paired with items from the 80 and 90% loss avoidance and 80% gain learning pairs). Participants were asked to choose the stimulus in each pair that they believed was “best” based on the first task phase. No feedback was provided during this phase. Following (Barch et al., 2017; Gold et al., 2012), pairings that allowed for differentiation of prediction error signaling and value representation were of greatest interest (see Supplementary Table 1).

The Brief Negative Symptom Scale (BNSS; Kirkpatrick et al., 2011) Clinical High-Risk Adaptation Version (Strauss & Chapman, 2018) was used to assess negative symptoms. The BNSS is a 13-item clinical interview-based rating scale of negative symptoms that covers the 5 NIMH consensus conference domains of anhedonia, avolition, asociality, blunted affect, and alogia. Based on confirmatory factor analysis (Strauss et al., 2018), the standard 5 domain scores were computed using averages of the items comprising each domain. Internal consistency for the BNSS total score in this sample was .89 and .90 for anhedonia, .87 for avolition, .85 for asociality, .94 for blunted affect, and .97 for alogia. Interviews for clinical rating scales were administered by a licensed clinical psychologist (GPS) or examiners trained to reliability standards (>0.80) using gold-standard training videos created by the PI.

### Data analysis

To test hypotheses about learning from gains and losses, a linear mixed effect model was used to compare the learning phase trials on the PRLT between all CHR and CN. Group (CHR, CN), valence (gain, loss), probability (80/20%, 90/10%), and learning block (1–4) were fixed factors, and subject-specific intercepts were random factors. Significant interactions were followed up by pairwise Tukey posthoc contrasts using the emmeans package in R. To test hypotheses about rapid learning, a learning composite score was calculated (Block 4–Block 1 accuracy; higher scores indicate greater learning of the optimal stimulus from the last block to the first block). The learning composite was compared between groups with an independent samples *t*-test. Hypotheses for group differences on the four novel stimulus pairings in the transfer phase were examined using one-way ANOVAs. Finally, correlations examined associations between performance in both phases and motivational negative symptom domains. Guided by our hypotheses, we focused on associations with motivational negative symptoms with the average accuracy for the gain and loss-avoidance conditions in the learning phase and the transfer performance scores for the Frequent Winner versus Frequent Loss Avoidance pairings. Supplemental exploratory associations with additional task metrics (learning composite, valence score differences; additional transfer performance scores) and clinical outcomes (cross-sectional risk of conversion to psychosis, functioning, depression, expressive negative symptoms) are presented in the Supplementary Material. The Benjamini and Hochberg correction for multiple comparisons was applied to account for the false-discovery rate. Instances where significant effects did not survive correction for multiple comparisons are noted.

Since negative symptoms are a hybrid categorical-dimensional construct (Ahmed et al., 2015), in addition to the dimensional (correlation) analyses, negative symptom effects were also evaluated categorically across 3 groups: CN and then CHR with high or low negative symptoms using a median split with the BNSS total score (supplemental analyses were also conducted using the BNSS MAP scores).

## Results

### Full group analyses

**Figure 1.**
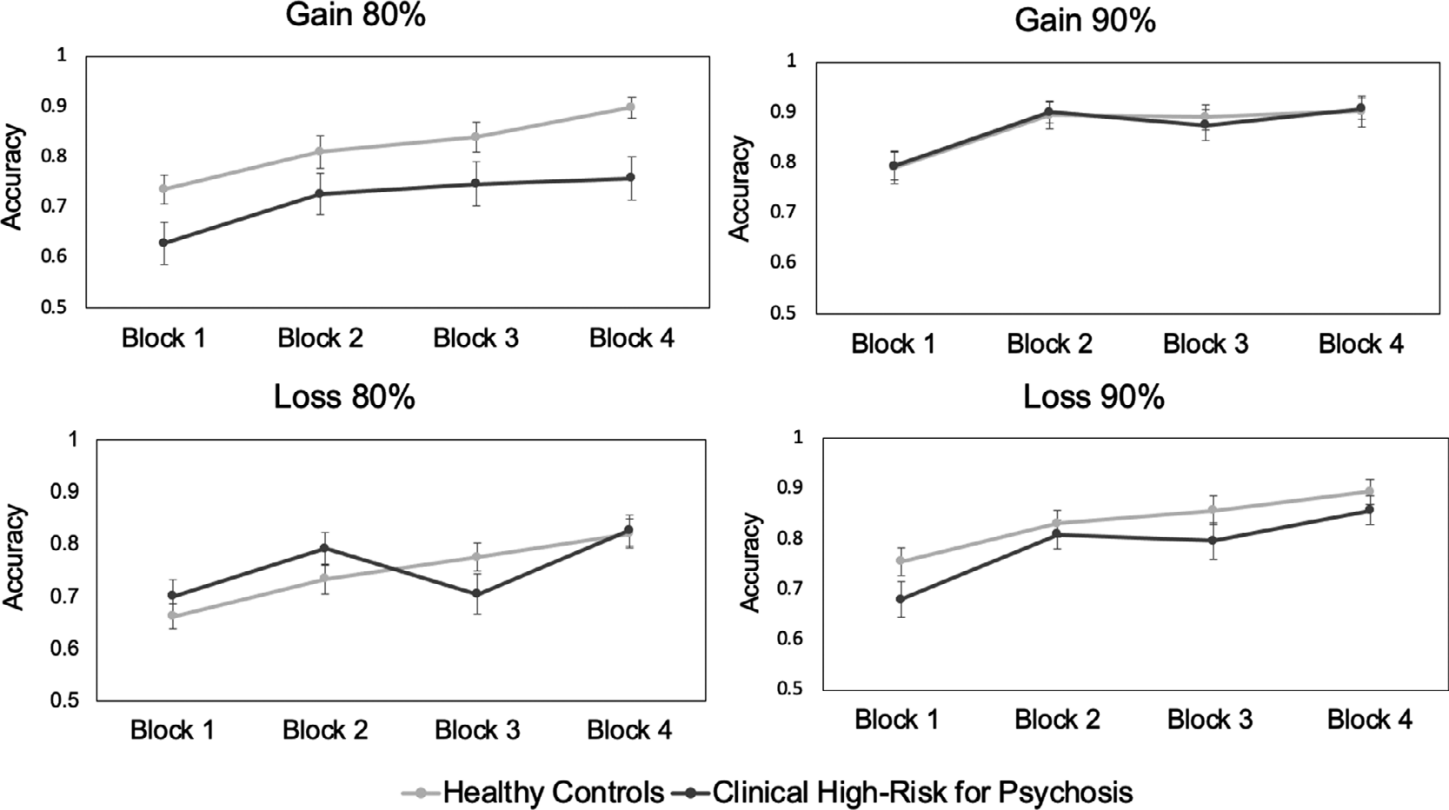
Training performance in healthy controls and clinical high-risk for psychosis samples. Mean accuracy is reported. Error bars denote the standard error of the mean (SEM).

*Acquisition phase.* The linear mixed effects model with all CHR and CN identified significant main effects of the block, probability, valence, and a trending (*p* = .10) main effect of group for predicting accuracy (see Table 2 and Figure 1). Post-hocs were used to interpret these effects. Post-hocs revealed that accuracy increased as probability increased (*p* < .01) and largely as block increased (*p*-values < .01, except blocks 2 and 3 did not differ, *p* = .99). Accuracy was significantly higher for gain than loss-avoidance conditions (*p* < .01).

**Table 2.** PRLT in CHR youth and healthy controls

	DF	F Value	*p*-value
Group	1,97	2.80	.097
Block	3,1455	37.40	< .001*
Probability	1,1455	69.91	< .001*
Valence	1,1455	15.40	< .001*
Block × probability	3,1455	.47	.70
Block × valence	3,1455	.67	.57
Probability × valence	1,1455	5.02	.02*
Group × block	3,1455	1.15	.33
Group × probability	1,1455	1.60	.21
Group × valence	1,1455	3.02	.08
Block × probability × valence	3,1455	.83	.48
Block × probability × group	3,1455	.49	.69
Block × valence × group	3,1455	.65	.58
Probability × valence × group	1,1455	18.18	<.001*
Block × probability × valence × group	3,1455	.77	.51

*Note*: CHR, clinical high-risk group; DF, degrees of freedom; PRLT, probabilistic reinforcement learning task.

There was also a significant interaction between probability and valence that was qualified by a significant probability X valence X group interaction. Post-hocs identified that this 3-way interaction was driven by CHR performing worse than CN in the 80% gain condition (*p* < .01). No significant group differences were observed for the other conditions. Thus, CHR displayed a selective deficit in learning from gains, which was driven by the 80% gain condition.

The overall learning composite did not significantly differ between CHR and CN (*p* = .82) (see Supplementary Table 2). In line with Gold et al. (2012), we also examined the impact of valence on learning by calculating difference scores between the final block for the gain and loss-avoidance trials for both the 90% and 80% pairs. Positive difference scores suggest increased gain learning, and negative difference scores indicate increased loss-avoidance learning. Independent sample t-tests comparing these scores (see Supplementary Table 2) demonstrated significant group differences on the 80% pairs (*t* = 2.69, p < .01); CN had better learning from gains than losses, and CHR demonstrated better learning from losses than gains. Groups did not significantly differ on 90% difference scores.

**Figure 2.**
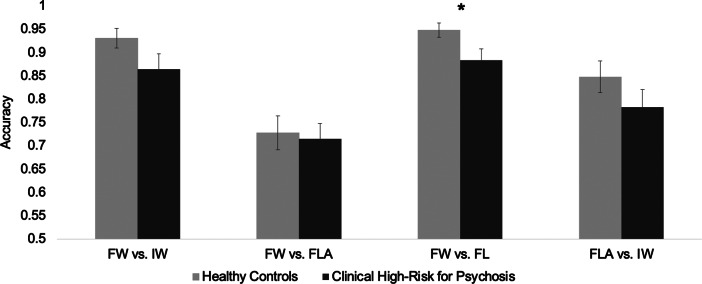
Transfer phase performance across groups. * *p* < .05. FLA, frequent loss avoider; FW, frequent winner; FL, frequent loser; IW, infrequent winner. Mean accuracy is graphed. Error bars denote the standard error of the mean (SEM). *p < .05.

*Transfer phase.* CHR chose stimuli associated with frequent winning over frequent losing (FW v. FL) significantly less than CN (*p* = .03) (see Figure 2). Groups did not significantly differ on choosing stimuli associated with frequent winning versus infrequent winning (FW v. IW) (*p* = .09), frequent winning versus frequent loss avoidance (*p* = .79) (FW v. FLA), or frequent loss avoidance versus infrequent winners (FLA v. IW) (*p* = .20).

*Correlations with Negative Symptoms.* In CHR, lower average gain learning was significantly associated with greater asociality (see Table 3). Average loss avoidance learning was not significantly associated with any negative symptoms. For the test phase, a reduced likelihood of choosing stimuli associated with FW versus FLA was significantly associated with greater asociality.

**Table 3.** Correlations between training and transfer performance and negative symptoms in the CHR group

	BNSS – Anhedonia	BNSS – Asociality	BNSS – Avolition
Training phase			
Average gain	.02	−.30*	.14
Average loss-avoidance	.27	−.23	.10
Test phase			
FW v FLA	−.20	−.34*	.04

The Benjamini-Hochberg procedure was used to correct for multiple comparisons. All noted significant correlations were retained. BNSS, Brief Negative Symptom Scale; FW v FLA, Frequent Winner versus Frequent Loss Avoider.

*Secondary Clinical Correlations.* Exploratory associations with depression, functioning, and risk of conversion to psychosis are presented in Supplementary Table 3. Most notably, the FW versus FLA findings extended to functioning and risk of conversion to psychosis, with a reduced likelihood of choosing stimuli associated with FW versus FLA being associated with a higher probability of conversion to a psychotic disorder and lower functioning. No significant associations were observed between training and transfer phase performance and depression, and correlations with negative symptoms were of similar magnitude after controlling for depression (see Supplementary Table 4).

### Analyses with CHR negative symptom subgroups

The results are briefly described below. See Supplementary Material, including Supplementary Table 5 and Supplementary Figure 2 for full results.

*Training Phase.* In the linear mixed effects model with high CHR negative symptoms, low CHR negative symptoms, and CN groups, a probability X valence X group interaction was observed. This was driven by group differences in gain learning on the 80% probability trials; the high negative symptom CHR group had poorer learning from gains than CN (*p* < .01).

The valence difference scores significantly differed between the three groups on the 80% pairs. The high negative symptom group learned worse from gains than losses compared to CN (*p* = .01).

*Transfer Phase.* There were significant group differences between choosing stimuli associated with FW v. FL (see Supplementary Figure 3). The high negative symptom CHR group showed the lowest preference for FW v. FL (versus CN; (*p* < .01)).

## Discussion

Negative symptoms are one of the earliest and strongest markers of risk for developing a psychotic disorder among CHR youth (Addington & Heinssen, 2012; Alderman et al., 2015; Demjaha et al., 2012; Healey et al., 2018). Therefore, the identification of mechanisms underlying negative symptoms in CHR youth could hasten the discovery of novel treatment targets for early intervention and prevention efforts. In PDs, explicit RL impairments have been identified as a reward-processing component that contributes to motivational negative symptoms (Strauss et al., 2014), including impairments in rapid RL and learning from gains (Gold et al., 2012; Reinen et al., 2014; Strauss et al., 2011; Waltz et al., 2007, 2011). It is unclear whether these RL processes are also associated with negative symptoms in CHR.

Our results largely supported hypotheses. Specifically, CHR showed: (1) impaired learning from gains; (2) intact loss avoidance learning; (3) impaired learning from gains was most consistently linked to greater negative symptom severity, whether measured continuously or categorically. Thus, similar to what has been observed in PDs (Gold et al., 2012; Reinen et al., 2014; Strauss et al., 2011, 2014; Waltz et al., 2007, 2011), negative symptoms were associated with a specific profile of RL impairment in CHR characterized by impaired learning from gains but not losses. However, in contrast to hypotheses and studies in PDs (Cicero et al., 2014; Waltz et al., 2007, 2011), CHR youth had a largely intact overall learning composite compared to CN. Decrements in some but not all domains of neurocognition have been observed in CHR youth at a smaller effect size than PDs (Catalan et al., 2021; Pratt et al., 2023). The lack of group differences in rapid learning may reflect another area of relatively preserved cognition in CHR. However, individual differences in functioning mirrored findings in the PD literature (Gold et al., 2012; Reinen et al., 2014; Waltz et al., 2007, 2011), as the overall learning composite was associated with worse functioning in supplemental analyses. Thus, impaired rapid RL may not characterize the majority of the CHR population but does occur in the subgroup with poor functioning.

The transfer phase—where stimuli were presented in novel pairings—helped to elucidate the nature of the identified deficits. Specifically, CHR youth in the whole sample and those with elevated negative symptoms showed a lower preference than CN for stimuli associated with frequently winning over frequently losing. In addition, greater negative symptoms were associated with less frequently choosing the stimuli associated with frequently winning over those associated with frequent loss avoidance. Since the stimuli within this transfer pair are associated with positive prediction errors during acquisition (i.e., gain reward or avoid losing reward, respectively), the selection of the frequent winner over the frequent loss avoider suggests that a participant can adequately represent reward value. This pattern is similar to how individuals with PDs perform (Gold et al., 2012). Our findings in conjunction with this past work in individuals with PDs, suggest these RL deficits begin prior to the onset of psychosis and are associated with negative symptoms transphasically (i.e., across illness phases). Specifically, negative symptoms are associated with difficulty representing reward value and using those representations to guide decision-making across phases of psychotic illness. These findings extend prior CHR studies examining value representation with other tasks (e.g., delay discounting), which also found associations with negative symptoms (Bartolomeo, Chapman, Raugh, & Strauss, 2021). Given evidence from past neuroimaging studies, these RL deficits may be driven by reduced activation of regions like the ventral striatum, anterior cingulate cortex, and ventromedial prefrontal cortex (Millman et al., 2020; Schmidt et al., 2017).

Supplemental results also indicated that no learning or transfer phase indices were linked to depression in CHR youth. Associations between task performance and negative symptoms, functioning, and cross-sectional risk of developing psychosis were also of similar magnitude after controlling for depression. This suggests our findings are not simply a result of depression or reflective of secondary processes that mask negative symptoms. The lack of an association with depression contrasts some prior CHR studies indicating aspects of reward processing (e.g., hedonic reactivity) are associated with greater depression severity (Strauss et al., 2018; Wotruba et al., 2014). Since secondary negative symptom processes, such as depression and anxiety, are highly prevalent in CHR (Fusar-Poli et al., 2014), additional studies with large samples are needed to clarify the role of depression in the observed associations.

A final important observation was that RL deficits were associated with a greater cross-sectional risk for developing a psychotic disorder and poorer functioning in secondary analyses; however, this may not be surprising since negative symptoms themselves are critically linked to both outcomes. RL deficits may serve as an intermediate phenotype that links neural functioning abnormalities (e.g., disrupted cortico-striatal connectivity) with negative symptoms and ultimately increased risk for conversion. Current efforts are underway through the CAPER consortium (Gold et al., 2020) to identify if performance on this task alone or in combination with other behavioral tasks assessing mechanisms of positive symptoms, disorganized symptoms, or other negative symptom mechanisms (e.g., effort-cost computation) may best predict conversion to psychosis in a longitudinal study. Identification of computerized tasks, such as the PRLT, that can provide a more objective marker of risk and require less training and clinical time than structured clinical interviews traditionally used to assess psychosis risk, may help to increase the scalability and accessibility of identifying CHR youth at greatest likelihood of converting.

Several limitations should be considered when interpreting these findings. First, the data reported is cross-sectional, and longitudinal data can more precisely identify the role of RL impairments in conversion to a psychotic disorder. Similarly, we cannot infer that RL deficits cause negative symptoms without a prospective longitudinal design that documents the initial emergence of negative symptoms and what predicts their onset/worsening over time. Second, the CHR sample was small and several of the associations were small to medium in magnitude; although the findings are bolstered by the fact that they largely align with prior PD studies using this paradigm (Barch et al., 2017; Gold et al., 2012) and other RL findings in CHR youth (Gold et al., 2012; Millman et al., 2020; Waltz et al., 2015), our findings should still be considered preliminary and replication in larger samples is needed to confirm the results. Future studies should also use a combination of functional neuroimaging and computational modeling approaches to confirm the presence of mechanisms that were inferred to be present based on behavioral performance alone. Further, although the BNSS version used was adapted for CHR youth (Strauss & Chapman, 2018), recently developed negative symptom measures for CHR youth, including the Negative Symptom Inventory for Psychosis Risk (NSI-PR; Strauss et al., 2023), may be more sensitive assessments in this population. Third, only a single reward processing task was administered. Given negative symptom models have posited that RL and other aspects of reward processing interact to produce motivational symptoms (Kring & Barch, 2014; Strauss et al., 2014), future studies could investigate whether the observed impairments are primary or secondary to other reward-processing processes in CHR (e.g., value representation, and hedonic deficits). Our findings are also limited to reinforcement learning in the context of monetary rewards, and additional work is needed to see if CHR youth have differential response patterns to monetary rewards versus other types of reward (social, food, etc), as not all reward types are encoded similarly (Simon et al., 2015; Ulrich et al., 2023; Wang, Liu, & Shi, 2020). Finally, given that neurocognitive functioning, especially working memory, has been shown to influence RL in adults with PDs (Barch et al., 2017; Collins et al., 2014 & Collins et al., 2017), future work is needed to more systematically examine how working memory capacity may interact with RL processes to influence negative symptoms in CHR youth.

With replication, there are several study implications. Most critically, these results suggest that impairments in positive reward learning and specifically effectively using value representations to guide decision-making may be a trans-phasic mechanism underlying negative symptoms in the PD-spectrum. It may therefore be beneficial to develop interventions targeting these mechanisms. For example, just-in-time mobile interventions (Nahum-Shani et al., 2018) could be used to promote engagement in goal-directed activities and actively encode and consolidate reward-related outcomes that can serve as future motivation. Indeed, mobile interventions using similar techniques to target value representation in SZ have shown initial efficacy in improving motivation in SZ (Luther et al., 2020). These interventions could also be bolstered through in-person therapies focused on using such techniques as behavioral experiments and Socratic questioning to support learning associations between engagement in novel activities and rewards. The PRLT may be an important endpoint in clinical trials to identify how and whether treatments are effectively engaging RL processes to improve negative symptoms and other aspects of psychosis symptomatology.

## Supporting information

Luther et al. supplementary material Luther et al. supplementary material

## Supplementary material

The supplementary material for this article can be found at http://doi.org/10.1017/S0033291724003416.
