# Supplementary material for: Probabalistic reinforcement learning impairments predict negative symptom severity and risk for conversion in youth at clinical high-risk for psychosis
Source: Psychol Med. 2025 Feb 6;55:e28. doi: 10.1017/S0033291724003416 (PMC12017368; doi:10.1017/S0033291724003416)
Supplement: Luther et al. supplementary material [file S0033291724003416sup001.docx]

**SUPPLEMENTAL MATERIALS FOR**

**PROBABALISTIC REINFORCEMENT LEARNING IMPAIRMENTS PREDICT NEGATIVE SYMPTOM SEVERITY AND RISK FOR CONVERSION IN YOUTH AT CLINICAL HIGH-RISK FOR PSYCHOSIS**

Lauren Luther, Ph.D.^a,b^

Ian M. Raugh, Ph.D.^b,c^

Gregory P. Strauss, Ph.D.^b^*

^a^ Department of Psychology, University of Alabama at Birmingham, Birmingham, AL, USA

^b^Department of Psychology, University of Georgia, Athens, GA, USA

^c^ Douglas Mental Health Institute, Department of Psychiatry, McGill University, Montréal, Québec, CA

*Correspondence concerning this article should be addressed to Gregory P. Strauss, Ph.D., Email: gstrauss@uga.edu. Phone: +1-706-542-0307. Fax: +1-706-542-3275. University of Georgia, Department of Psychology, 125 Baldwin St., Athens, GA 30602.

Supplemental Methods

Prodromal Syndrome Types in Clinical High-Risk (CHR) for Psychosis Sample

Attenuated Positive Symptoms (i.e., SIPS score of at least 3–5 on at least one positive symptom item; n = 44), Brief Intermittent Psychosis Syndrome (i.e., SIPS score of 6 on at least one positive symptom item, with symptoms present at least several minutes a day at a frequency of at least once per month; n = 1), or Genetic Risk and Deterioration Syndrome (i.e.,1st degree relative with a psychotic disorder and decline in global functioning over the past year) and Attenuated Positive Symptoms (n = 1). Thirty-seven CHR participants met criteria for progression, 8 for persistence, and 1 for partial remission.

| *Supplemental Table 1*. Key Parings in Transfer Phase | |
| --- | --- |
| Key Pairings in Transfer Phase | Constructs Assessed |
| Frequent Winner (FW) vs. Frequent Loss Avoider (FLA) | Sensitivity to gains versus loss avoidance |
| Frequent Winner (FW) vs. Infrequent Winner (IW) | Sensitivity to frequency of feedback about gains versus less frequent gains |
| Frequent Winner (FW) vs. Frequent Loser (FL) | Sensitivity to gain versus loss |
| Frequent Loss Avoider (FLA) vs. Infrequent Winner (IW) | Sensitivity to frequent loss avoidance versus less frequent gains |

Based on Barch et al (2017).

| *Supplemental Table 2.* Descriptive Statistics for Learning rate and Valence Learning Difference Scores | | | | |
| --- | --- | --- | --- | --- |
|  | Clinical-High Risk for Psychosis | Healthy Controls | F | P |
|  | M (SD) | M (SD) |  |  |
| Learning Rate | .54 (.73) | .57 (.55) | .05 | .82 |
| Valence Learning Differences 80% | -.07 (.32) | .08 (.22) | 7.23 | .008 |
| Valence Learning Differences 90% | .05 (.19) | .01 (.26) | .81 | .37 |

Note. M = Mean; SD = standard deviation.

*Supplemental Table 3*. Full correlation matrix between training and transfer performance and clinical measures in the CHR group

|  | BNSS – Anhedonia | BNSS – Asociality | BNSS – Avolition | BNSS – Blunted affect | BNSS – Alogia | GAF – Functioning | SIPS - Depression | SHARP Risk Calculator |
| --- | --- | --- | --- | --- | --- | --- | --- | --- |
| Training Phase |  |  |  |  |  |  |  |  |
| Gain 80% | .03 | -.29 | .13 | .01 | -.08 | -.04 | .05 | -.04 |
| Gain 90% | .00 | -.13 | .10 | -.06 | -.12 | .33^*^ | -.11 | .07 |
| Average Gain | .02 | -.30* | .14 | -.01 | -.20 | .10 | .01 | -.01 |
| Loss-Avoidance 80% | .14 | -.16 | .02 | .05 | .25 | .08 | .11 | -.02 |
| Loss-Avoidance 90% | .32* | -.15 | .18 | .12 | .04 | .03 | -.12 | .07 |
| Average Loss-Avoidance | .27 | -.23 | .10 | .11 | .17 | .00 | -.02 | .07 |
| Learning Composite | .12 | -.26 | -.15 | .22 | .10 | .32* | -.22 | .24 |
| Valence Learning Differences 80% | -.12 | -.28 | .04 | -.06 | -.11 | .02 | .06 | -.06 |
| Valence Learning Differences 90% | -.16 | .13 | .10 | -.27 | -.21 | .13 | .07 | -.04 |
| Valence Learning Differences Average | -.10 | -.14 | .15 | -.14 | -.19 | .05 | .08 | -.05 |
| Test Phase |  |  |  |  |  |  |  |  |
| FW v IW | -.22 | -.20 | .18 | .12 | .05 | -.03 | .14 | -.08 |
| FW v FLA | -.20 | -.34* | .04 | -.11 | -.09 | .38^**^ | -.06 | -.32^*^ |
| FW v FL | .01 | -.26 | .00 | -.10 | -.14 | .18 | -.12 | .08 |
| FLA v IW | .05 | -.08 | .30* | .02 | -.16 | -.08 | .08 | .17 |

Note. ** p < .01, * p < .05. Valence learning difference scores are final block for the gain minus final block for loss-avoidance trials. Positive difference scores suggest increased learning from gains, and negative difference scores indicate increased learning from loss-avoidance. BNSS = Brief Negative Symptom Scale; FLA = frequent loss avoider; FW = frequent winner; FL = frequent loser; GAF = Global Assessment of Functioning Scale; IW = infrequent winner; SIPS = Structured Interview for Psychosis-Risk Syndromes Scale; SHARP = Shanghai at Risk for Psychosis.

*Supplemental Table 4*. Correlations between training and transfer performance and clinical measures in the CHR group after controlling for depression

|  | BNSS – Anhedonia | BNSS – Asociality | BNSS – Avolition | GAF – Functioning | SHARP Risk Calculator |
| --- | --- | --- | --- | --- | --- |
| Training Phase |  |  |  |  |  |
| Gain 80% | .02 | -.20 | .16 | .08 | .01 |
| Gain 90% | .08 | -.04 | .19 | .29 | .05 |
| Loss-Avoidance 80% | .17 | -.15 | .01 | .14 | -.08 |
| Loss-Avoidance 90% | .28 | .04 | .28 | -.09 | .18 |
| Learning Rate | .2 | -.20 | .07 | .26 | .14 |
| Valence Learning Differences 80% | -.13 | -.31* | .02 | .13 | .05 |
| Valence Learning Differences 90% | -.06 | .05 | .08 | .17 | -.12 |
| Test Phase |  |  |  |  |  |
| FW v IW | -.19 | -.11 | .17 | .07 | -.10 |
| FW v FLA | -.19 | -.35* | .11 | .47** | -.31* |
| FW v FL | -.01 | -.33* | .00 | .22 | .00 |
| FLA v IW | .00 | .04 | .36* | -.08 | .17 |

Note. ** p < .01, * p < .05. Valence difference scores are final block for the gain minus final block for loss-avoidance trials. Positive difference scores suggest increased learning from gains, and negative difference scores indicate increased learning from loss-avoidance. BNSS = Brief Negative Symptom Scale; CHR = clinical-high risk for psychosis group; FLA = frequent loss avoider; FW = frequent winner; FL = frequent loser; IW = infrequent winner.

Supplemental Results

*Analyses with CHR negative symptom subgroups.*

*Training Phase.* Analyses identified main effects of block, probability, valence (see Supplemental Table 5; supplemental Figure 2). No main effect of group was observed (*p* = .13). Similar to the cross-sectional CHR model, accuracy improved as probability increased from 80 to 90% (*p* = .0001), on trials associated with learning from gains rather than losses (p = .002), and largely as block increased (all p-values < .002; except block 2 versus 3, p = .84).

Several interactions were observed that qualified these main effects: 1) probability X valence, 2) block X group, and 3) probability X valence X group. The source of the probability X valence X group interaction was driven by group differences in the gain learning condition on the 80% probability trials: The high negative symptom CHR group had significantly lower accuracy than CN in the gain learning condition in the 80% probability trials (p < .0001). Groups did not significantly differ in any of the remaining gain conditions (p > .10) or loss avoidance conditions (p-value’s > .20). We also conducted these analyses with the BNSS MAP dimension (see Supplemental Table 6); results were highly similar, with the probability X valence X group integration being largely driven once again by the high negative symptom CHR group performing significantly worse than CN in the 80% gain learning condition (p = .0002).

Examining group differences on the valence difference scores with a one-way ANOVA suggested that the three groups significantly differed on the 80% pairs (F(2, 93)= 3.96, p = .02) but not 90% pairs (F(2, 93)= .46, p = .64). Post-hoc LSD contrasts identified that the high negative symptom group learned worse from gains than loss-avoidance than the CN group (p = .01).

*Transfer Phase.* There were significant group differences between choosing stimuli associated with frequently winning versus frequently losing (F(2, 93)= 4.05, *p* = .02) (see supplemental Figure 3), with the high negative symptom group showing the lowest preference for frequent winners over frequent loss avoiders; this was significantly lower than CN (*p* = .005).

| *Supplemental Table 5*. PRLT in CHR youth with high and low negative symptoms and Healthy Controls – BNSS Total | | | |
| --- | --- | --- | --- |
|  | DF | F Value | p-value |
| Group | 2,96 | 2.11 | .13 |
| Block | 3,1440 | 30.96 | < .001* |
| Probability | 1,1440 | 65.22 | < .001* |
| Valence | 1,1440 | 9.93 | .002* |
| Block X Probability | 3,1440 | .43 | .73 |
| Block X Valence | 3,1440 | 1.00 | .39 |
| Probability X Valence | 1,1440 | 11.46 | < .001* |
| Group X Block | 6, 1440 | 2.40 | .03* |
| Group X Probability | 2,1440 | 1.43 | .24 |
| Group X Valence | 2,1440 | 2.59 | .08 |
| Block X Probability X Valence | 3, 1440 | 1.17 | .32 |
| Block X Probability X Group | 6, 1440 | .76 | .60 |
| Block X Valence X Group | 6, 1440 | 1.17 | .32 |
| Probability X Valence X Group | 2, 1440 | 9.87 | < .001* |
| Block X Probability X Valence X Group | 6, 1440 | .48 | .82 |

Note. BNSS = Brief Negative Symptom Scale; CHR = clinical-high risk for psychosis group; DF = degrees of freedom; PRLT = Probabilistic Reinforcement Learning Task.

| *Supplemental Table 6*. PRLT in CHR youth with high and low negative symptoms and Healthy Controls – BNSS MAP | | | |
| --- | --- | --- | --- |
|  | DF | F Value | p-value |
| Group | 2,96 | 1.63 | .20 |
| Block | 3,1440 | 31.19 | < .001* |
| Probability | 1,1440 | 64.36 | < .001* |
| Valence | 1,1440 | 9.69 | .002* |
| Block X Probability | 3,1440 | .41 | .74 |
| Block X Valence | 3,1440 | .94 | .42 |
| Probability X Valence | 1,1440 | 11.33 | < .001* |
| Group X Block | 6, 1440 | .83 | .55 |
| Group X Probability | 2,1440 | 1.83 | .16 |
| Group X Valence | 2,1440 | 2.08 | .13 |
| Block X Probability X Valence | 3, 1440 | 1.20 | .31 |
| Block X Probability X Group | 6, 1440 | .53 | .79 |
| Block X Valence X Group | 6, 1440 | .52 | .79 |
| Probability X Valence X Group | 2, 1440 | 9.88 | < .001* |
| Block X Probability X Valence X Group | 6, 1440 | .86 | .52 |

Note. BNSS = Brief Negative Symptom Scale; CHR = clinical-high risk for psychosis group; MAP = Motivation and pleasure factor score; PRLT = Probabilistic Reinforcement Learning Task.

*Supplemental Figure 1*. Example Trials from Probabilistic Reinforcement Learning Task

1. Trial Pairs Associated with Potential Gains

**Example Image Pair**


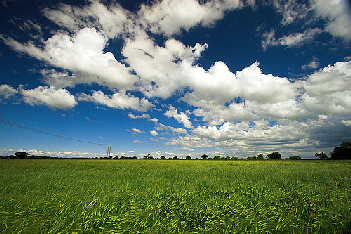

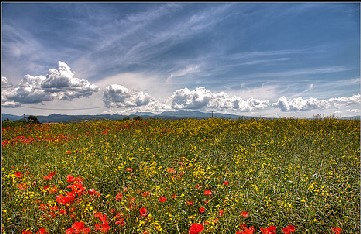


**Feedback**

Incorrect Response

Correct Response


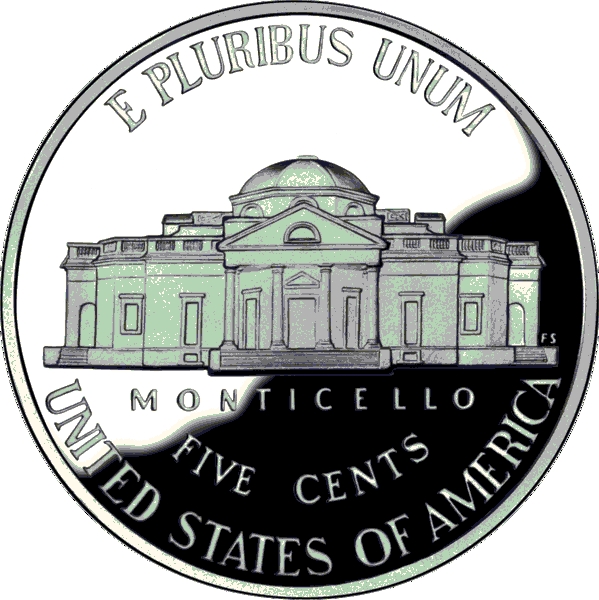


Not a winner. Try again!

Win!

1. Trial Pairs Associated with Loss Avoidance

**Example Image Pair**


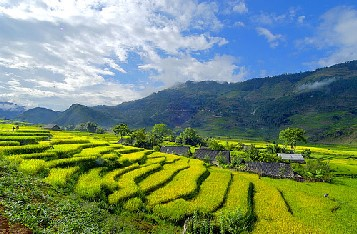

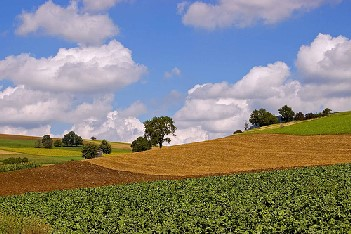


**Feedback**

Correct Response

Incorrect Response


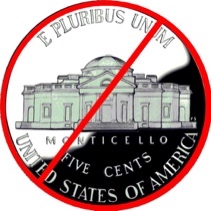


Lose!

Keep your money!


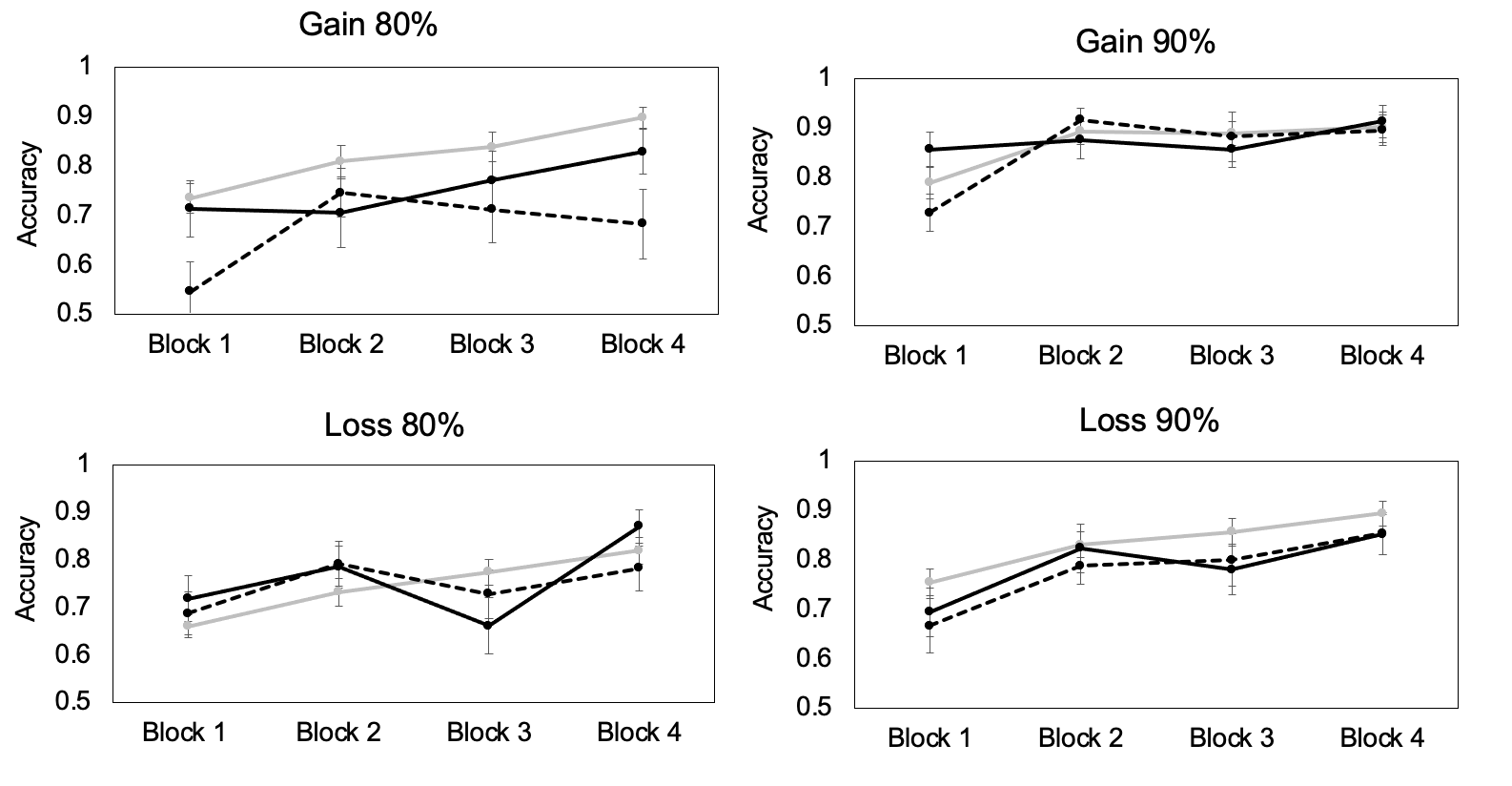


**
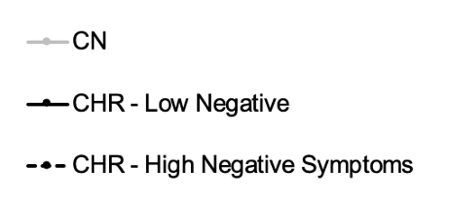
**

*Supplemental Figure 2.* Training Performance in Healthy Controls and Clinical High-Risk for Psychosis Negative Symptom Subgroups. Mean accuracy is graphed. Error bars denote standard error of the mean (SEM). CHR = clinical high risk for psychosis; CN = healthy controls.

**
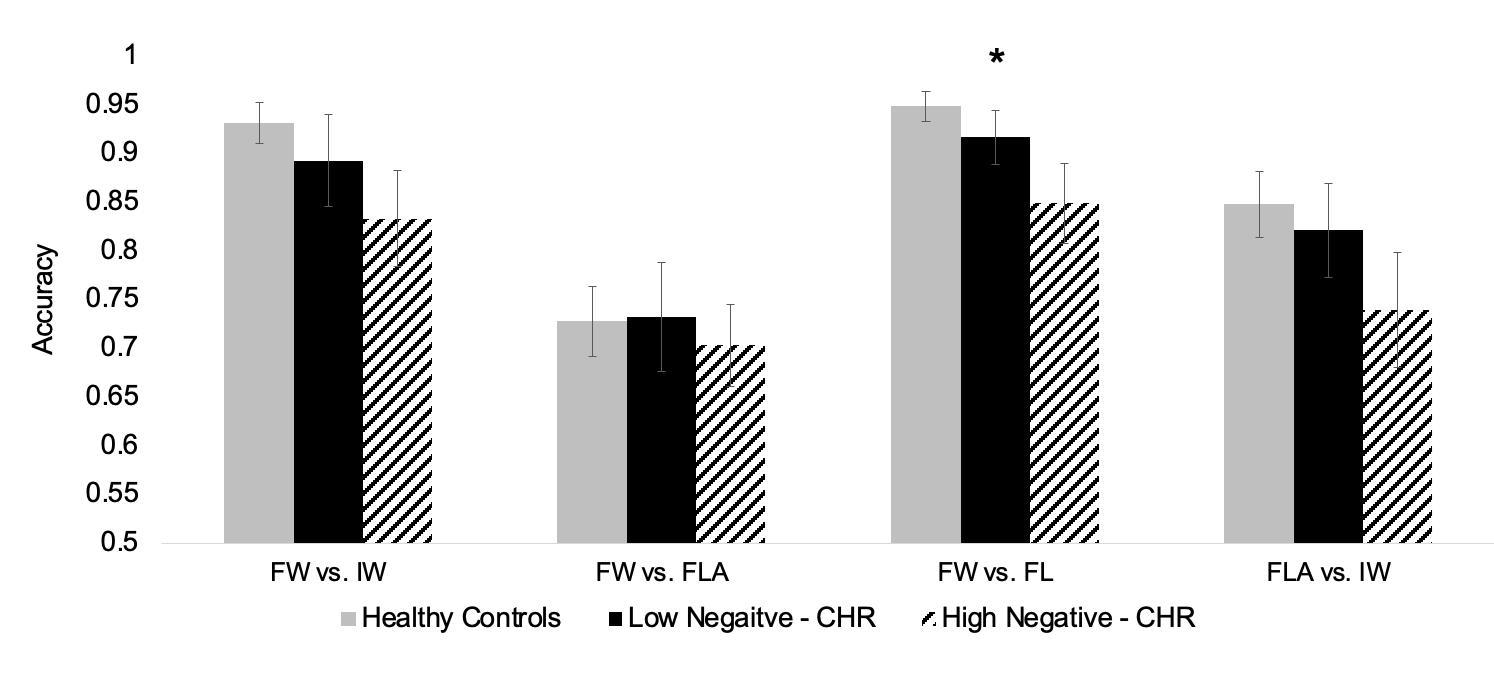
**

*Supplemental Figure 3.* Transfer Phase Performance Across Healthy Controls and Clinical High-Risk (CHR) for Psychosis Negative Symptom Groups with BNSS Total. * p < .05. BNSS = Brief Negative Symptom Scale; FLA = frequent loss avoider; FW = frequent winner; FL = frequent loser; IW = infrequent winner. Mean accuracy is graphed. Error bars denote standard error of the mean (SEM).
